# Survival outcomes and risk factors for liver and pancreatic metastases in renal cell carcinoma after curative nephrectomy

**DOI:** 10.1186/s12894-025-01802-x

**Published:** 2025-05-15

**Authors:** Kristina Hasselgren, Ali Bencherki, Jennifer Short, Anna Bendler, Yumer Mehriban, Mats Fredrikson, Per-Olof Lundgren, Martin Holmbom

**Affiliations:** 1 Department of Surgery in Linköping, Department of Biomedical and Clinical Sciences, Linköping University, Linköping, Sweden; 2 Department of Urology, Skaraborg Hospital, Skövde, Sweden; 3 Department of Urology in Östergötland, Department of Biomedical and Clinical Sciences, Linköping University, Linköping, 581 85 Sweden; 4 Department of Radiology in Linköping, Department of Health, Medicine and Caring Sciences, Linköping University, Linköping, Sweden; 5 Department of Clinical Pathology, Department of Biomedical and Clinical Sciences, Linköping University, Linköping, Sweden; 6 Department of Biomedical and Clinical Sciences and Forum Östergötland, Faculty of Medicine and Health Sciences, Linköping, Sweden; 7 Department of Urology, Karolinska Institute, Karolinska University Hospital, Stockholm, 141 86 Sweden

**Keywords:** Liver and pancreatic metastasis, Metastasis, RCC, Renal cell carcinoma, Risk factors, Survival

## Abstract

**Background:**

Renal cell carcinoma (RCC) has a high recurrence risk, with 20–40% of patients developing metastatic disease post-nephrectomy. This study aimed to identify risk factors associated with liver and pancreatic metastases in patients who have previously undergone curative nephrectomy for RCC.

**Methods:**

This retrospective cohort study with a nested case-control design included adults who underwent nephrectomy for non-metastatic RCC (non-mRCC) between 2009 and 2021. Patients who developed liver or pancreatic metastases, confirmed by secondary surgery, formed the case group. A randomly selected control group of non-mRCC patients were included to assess risk factors. Clinical, radiological, and pathological data were analyzed.

**Results:**

Among 967 nephrectomy patients, 754 (78%) had RCC, and 6% developed liver or pancreatic metastases. Advanced tumor stage (T3) was a significant predictor of RCC metastasis in these patients. Patients with a prolonged disease-free interval demonstrated better surgical eligibility and survival outcomes. Median time from nephrectomy to metastasis was 57 months in surgical cases compared to 22 months in non-surgical cases. Notably, 92% of patients eligible for metastasis surgery had clear cell RCC (ccRCC). Surgical resection was associated with improved survival, with 1-, 3-, and 5-year survival rates of 92%, 83%, and 75%, respectively, compared to 77%, 65%, and 56% for non-surgical patients.

**Conclusions:**

Advanced tumor stage and local invasiveness were key predictors of liver and pancreatic metastases in RCC. Prolonged time to metastasis improved surgical eligibility and survival. The majority of patients eligible for metastasis surgery had ccRCC. Further studies are needed to evaluate whether early, individualized follow-up for high-risk RCC subtypes improved surgical eligibility for liver and pancreatic metastasis.

**Supplementary Information:**

The online version contains supplementary material available at 10.1186/s12894-025-01802-x.

## Background

Renal cell carcinoma (RCC) is a significant health concern, with approximately 1,300 new cases diagnosed annually in Sweden. In 2020, the incidence was reported as 14.2 per 100,000 males and 5.7 per 100,000 females, with the disease accounting for nearly 500 deaths yearly in Sweden [1, 2]. The prognosis for RCC varies significantly depending on tumor stage at diagnosis. When RCC is confined to the kidney (stage I), the 5-year survival rate can reach as high as 95% [3]. Surgical resection remains the cornerstone of curative treatment for localized RCC. However, despite complete oncological resection, a substantial proportion of patients estimated between 20% and 40%—will develop metastatic disease. Notably, approximately 10% of patients present with metastases more than five years post-nephrectomy, indicating a risk of late metastatic recurrence. Liver metastases are common sites of RCC metastasis and are associated with a significant reduction in the 5-year survival rate, decreasing to 10-20% [4–7]. In contrast, pancreatic metastases are relatively rare, with an incidence ranging from 1.6 to 11% in patients with advanced RCC. Nevertheless, the pancreas is a notable location for metastatic spread for RCC, often representing as an isolated metastasis and typically occurring several years following an initial nephrectomy [8–11]. Given the distinct metastatic patterns and survival outcomes associated with liver and pancreatic metastases, identifying risk factors or prognostic indicators following curative-intent treatment of RCC is crucial for improving patient management and outcomes.

This study aimed to identify risk factors associated with the development of liver or pancreatic metastases following curative-intent treatment of RCC.

## Methods

### Study design

This retrospective cohort study with a nested case-control design investigated patients who underwent nephrectomy for RCC without evidence of metastasis (RCC-M0) at the time of surgery. Patients were selected from a comprehensive cohort in a Swedish county with a population of approximately 460 000 inhabitants, who were treated with nephrectomy between 2009 and 2021. Eligible patients included all adults (≥ 18 years) with a diagnosis of RCC, identified through database searches using International Classification of Diseases (ICD) and Swedish Classification of Medical Procedures (KVÅ) codes relevant to nephrectomy and RCC (KAC00, KAC20, KAD00, KAC21, TKA00, KAW96). Additional ICD codes for malignancies, specifically hepatic or pancreatic metastases (C78.0, C78.7, C79.9, C79.5, C77.0, C26.0, C77.1), were used to identify those with secondary metastatic disease. From an initial cohort of primary nephrectomy procedures, a subset of patients who developed metastases to the liver or pancreas was identified. After excluding inoperable cases and those without confirmed RCC post-surgery, a group of patients who underwent surgical intervention for RCC metastases was selected. To ensure that only RCC metastases were included, we excluded patients with other types of metastases. This resulted in a smaller subset of patients (*n* = 12) who underwent metastasectomy. To evaluate the risk profile for metastasis, a control group of 24 RCC patients without evidence of metastasis (N0M0) at time of nephrectomy or during follow-up was established. Control patients were randomly selected from the original cohort of patients who underwent primary nephrectomy between 2009 and 2021 and were not matched to the cases. To evaluate survival after metastatic surgery, patients who underwent surgery were compared with those who did not. No additional treatments, including adjuvant therapy were available during the study period. All surgeries and pathological evaluations were conducted at a single centre, Linköping university hospital.

### Data collection

Data was collected through a systematic review of electronic medical records. Key variables included demographic, clinical, radiological, and pathological data, as well as information on comorbid conditions, surgical outcomes, and survival.

*Demographic Variables*: Age, sex, body mass index (BMI), smoking status, and alcohol consumption at primary surgery. *Clinical Characteristics*: These included the American Society of Anesthesiologists (ASA) score (= 2), the Eastern Cooperative Oncology Group (ECOG) performance status (= 2), presence of local symptoms, hemoglobin (Hb) levels, platelet and neutrophil counts, and the update Charlson Comorbidity Index (CCI) [12]. *Radiological Characteristics*: Tumor localization (right or left kidney), dimensions (length and width), presence of venous invasion, collecting system invasion, perinephric and sinus fat invasion, and adrenal involvement. *Tumor and Pathological Characteristics*: TNM classification (T, N, and M staging), tumor grade, lymphovascular invasion, presence of tumor necrosis, sarcomatoid differentiation, and radical resection status. *Metastatic Characteristics and Surgical Interventions*: For the case group, data on liver and pancreatic metastases were collected, including size, number, and specific characteristics of each metastasis. Information regarding secondary surgical interventions (e.g., liver resection, Whipple procedure, left-sided pancreatectomy) was also documented.

### Follow-up

Time intervals from primary nephrectomy to the development of liver or pancreatic metastases were recorded, along with all-cause mortality rates. For patients undergoing secondary surgical intervention, time from primary to secondary surgery, as well as from secondary surgery to mortality, was documented. The median follow-up period for both groups was calculated to assess survival outcomes at 1, 3 and 5 years.

### Statistical analysis

Descriptive statistics were applied to summarize demographic, clinical, radiological, and pathological data. For univariable analysis, Chi-square tests, t-tests, or non-parametric equivalents were utilized to identify potential factors associated with the development of liver or pancreatic metastases. Significant variables from the univariable analysis were further evaluated in a multivariable logistic regression model to determine their independent association with metastatic development. Odds ratios (ORs) with 95% confidence intervals (CIs) were calculated for each variable, and statistical significance was defined as *p* < 0.05. A bootstrap resampling method with 5,000 iterations was applied to the logistic regression model to validate the robustness of significant variables. In each iteration we randomly sampled the dataset with replacement, recalculated the regression model and assessed stability of the estimated coefficients for the predictors. The resampling process involved the inclusion of all predictors from the multivariable logistic regression model. Bias-corrected standard errors and adjusted 95% CI were calculated for each predictor.

## Results

A total of 967 patients who underwent nephrectomy between 2009 and 2021 were included in the study, of whom 754 (78%) were diagnosed with RCC. Of these, 236 patients (31%) developed a secondary malignancy, with 48 (6%) presenting with liver or pancreatic metastases. Twelve patients met the inclusion criteria for RCC metastasis to the pancreas (*n* = 8) or liver (*n* = 4), forming the case group. This group was compared to a control group of 24 patients without liver or pancreatic metastases.

### Demographics and clinical characteristics

Demographic characteristics showed no significant differences between the case and control groups. The proportion of male patients was 83% (*n* = 10) in the case group and 63% (*n* = 15) in the control group (*p* = 0.374). The mean age at the time of nephrectomy was 64 years in the case group and 66 years in the control group (*p* = 0.629). Body mass index (BMI) was similar between groups (26 vs. 27; *p* = 0.470), and no significant differences in smoking or alcohol consumption were observed (*p* = 0.999 and *p* = 0.721, respectively).

Comorbidities, as measured by the Charlson comorbidity index, showed a median score of 3.0 in the case group and 2.5 in the control group (*p* = 0.280), with no significant differences in the incidence of myocardial infarction, congestive heart failure, or other conditions included in the study (Table 1).

**Table 1:** Comparison of demographic, clinical, radiological, and pathological characteristics between patients with and without liver or pancreatic metastasis following primary surgery for renal cell carcinoma

	Case, n12	Control, n24	*P*-value
Male	10 (83)	15 (63)	0.268
Age ^a^	64 (10)	66 (8)	0.629
BMI ^a^	26 (3)	27 (3)	0.469
Smoking	2 (17)	5 (21)	> 0.999
Alcohol	6 (50)	9 (38)	0.721
ASA ≥ 2	1 (8)	1 (4)	> 0.999
ECOG ≥ 2	0 (0)	1 (4)	> 0.999
Local symptoms	8 (67)	10 (42)	0.289
Hb ^a^	131 (14)	133 (14)	0.674
Platelet count ^a^	287 (56)	294 (58)	0.734
Neutrohil count ^b^	8.4 (8–9)	8.5 (8–9)	0.168
Charlson score ^a^	3.0 (1.5)	2.6 (0.8)	0.284
**Comorbidity**			
Mycardial infarction	2 (17)	3 (13)	> 0.999
Congestive heart failure	2 (17)	4 (17)	> 0.999
Peripheral vascular disease	0 (0)	0 (0)	-
Cerebovascular disease	2 (17)	2 (8)	0.815
Dementia	0 (0)	0 (0)	-
Chronic pulmonary disease	0 (0)	1 (4)	> 0.999
Connective tissue disease	0 (0)	0 (0)	-
Ulcer disease	4 (33)	5 (21)	0.670
Mild liver disease	0 (0)	0 (0)	-
Diabetes type I and II	4 (33)	2 (8)	0.161
Hemiplegia	0 (0)	0 (0)	-
Moderate to severe renal disease	4 (33)	5 (21)	0.161
Any tumor	12 (100)	24 (100)	-
Moderate to severe liver liver disease	0 (0)	0 (0)	-
Metastatic solid tumor	0 (0)	0 (0)	-
**Radiology**			
Right	5 (42)	11 (46)	0.813
Left	7 (58)	13 (54)	0.813
Tumor size length (mm) ^a^	72 (33)	78 (29)	0.979
Tumor size width (mm) ^a^	58 (25)	67 (24)	0.855
Venous invasion and extension	4 (33)	3 (13)	0.298
Collecting system invasion	8 (67)	4 (17)	*0.009*
Perinephric and sinus fat invasion	10 (83)	9 (38)	*0.009*
Adrenal involment	0 (0)	0 (0)	-
**TNM**			
T1a-T1bN0M0	0 (0)	7 (29)	*0.070*
T2a-T2bN0M0	0 (0)	7 (29)	*0.070*
T3a-T3bN0M0/x	11 (92)	9 (38)	*< 0.001*
T3aN0M0	4 (33)	4 (17)	0.397
T3aN0Mx	5 (42)	3 (13	0.086
T3bN0M0	2 (17)	2 (8)	0.588
T4N0M0	1 (8)	1 (4)	> 0.999
**Intervention**	12 (100)	24 (100)	-
Nephrectomy	10 (83)	17 (71)	0.685
Partial nephrectomy	2 (17)	7 (29)	0.685
Ablativa treatment	0 (0)	0 (0)	-
**Pathology**			
Tumour grade 1	0 (0)	3 (13)	0.284
Tumour grade 2	8 (67)	18 (75)	0.880
Tumour grade 3	2 (17)	2 (8)	0.815
Tumour grade 4	2 (17)	1 (4)	0.505
**Subtypes**			
Clear Cell (ccRCC)	11 (92)	19 (79)	0.400
Papillary (pRCC)	0 (0)	3 (13)	0.284
Chromophobe (chRCC)	1 (8)	2 (8)	> 0.999
Lymphovascular invasion	3 (25)	3 (13)	0.378
Tumour necrosis	4 (33)	3 (13)	0.190
Invasion of the collecting system	2 (17)	2 (8)	0.588
Sarcomatoid changes	2 (17)	2 (8)	0.588
Rhabdoid changes	0 (0)	0 (0)	-
Radical resection	12 (100)	24 (100)	-

ASA = American Society of Anesthesiologists; ECOG = Eastern Cooperative Oncology Group; TNM = tumor, node, metastasis; ccRCC = Clear Cell Renal Cell Carcinoma, pRCC = Papillary Renal Cell Carcinoma, chRCC = Chromophobe Renal Cell Carcinoma; ^a^ Mean value (SD); ^b^ Median value (IQR)

### Radiological and tumor characteristics

Radiologically, tumor localization was similar in both groups, with no significant differences between right- vs. left-sided tumors (42% vs. 46%, *p* = 0.813). Tumor size showed no significant difference between groups, with median length of 72 mm in the case group and 78 mm in the control group (*p* = 0.813). However, significant differences in local tumor aggressiveness was observed. The case group exhibited significantly higher rates of collecting system invasion (67% vs. 17%; *p* = 0.009) and perinephric or sinus fat invasion (83% vs. 38%; *p* = 0.009) (Table 1).

### TNM staging

Advanced tumor stage was more common in the case group, with 92% of patients classified as T3a-T3bN0M0/x, compared to 38% in the control group (*p* < 0.001). None of the case group patients had T1 or T2 disease, reinforcing the association between advanced staging (T3) and the development of liver or pancreatic metastases (Table 1).

### Pathological features

Tumor grade distribution did not significantly differ between the two groups, although 67% of case group patients had grade 2 tumors. Tumor necrosis was more prevalent in the case group (33% vs. 13%; *p* = 0.190), though this difference did not reach statistical significance. No significant differences were observed in lymphovascular invasion, sarcomatoid changes, or other pathological features (Table 1). In the case group, 92% of patients had clear cell RCC (ccRCC), 8% chromophobe RCC (chRCC), and 0% papillary RCC (pRCC). In comparison, the control group consisted of 78% ccRCC, 14% pRCC and 8% chRCC.

### Surgical and metastatic outcomes

In the case group (*n* = 12), liver metastases were present in 4 patients (33%), with a median lesion size of 19 mm and 2.5 metastases. All were treated with radical resection. Pancreatic metastases occurred in 8 patients (67%), with a median lesion size of 25.5 mm and 2 metastases. Surgical interventions included Whipple procedure (38%), left-sided pancreatectomy (50%), and total pancreatectomy (13%). The median time from primary nephrectomy to secondary surgery was 56 months (Table 2).

**Table 2:** Metastasis of renal cell carcinoma to the liver or pancreas: surgical interventions, tumor characteristics, and survival outcomes

Metastasis of Renal Cell Carcinoma to the Liver or Pancreas	n12
**Liver**	4 (33)
Size (mm) ^a^	24 (15)
Number of metastases ^a^	2 (1)
Radical	4 (100)
***Localisation***	
3,4b/5,7	1 (25)
4b, 5, 6	1 (25)
8	1 (25)
1	1 (25)
***Type of intervention***	
Resection	4 (100)
**Pancreas**	8 (67)
Size (mm) ^a^	26 (6)
Number of metastases ^a^	2 (1)
Radical	8 (100)
***Localisation***	
Caput	4 (50)
Cauda	3 (38)
The whole pancreas	1 (13)
***Type of intervention***	
Whipple	3 (38)
Left-sided pancreatectomy	4 (50)
Pancreatectomy	1 (13)
**Time from primary surgery to secondary surgery (months)** ^b^	56
**All-cause mortality**	6 (50)

^a^ Mean value (SD); ^b^ Median value (IQR)

### Multivariable logistic regression and bootstrap analysis

Multivariable analysis identified stage T3 as a significant predictor of liver or pancreatic metastasis, with an odds ratio (OR) of 41 (95% CI, 2.8–606; *p* = 0.002). Male gender and tumor necrosis showed elevated ORs (5.9 and 6.3, respectively), but did not reach statistical significance (*p* = 0.097 and *p* = 0.149). These findings nevertheless highlight the strong association between advanced tumor stage and the development of liver or pancreatic metastases in RCC patients (Table 3). Bootstrap analysis (5,000 samples) confirmed stage T3 as a robust predictor of metastasis, with a bootstrapped coefficient of 3.716 (95% CI, 1.942–55.426; *p* = 0.002). (Supplementary Table 1).

**Table 3:** Factors associated with liver or pancreas metastasis following primary surgery for renal cell carcinoma– Multivariate analysis

Variable	OR (95% CI)	*P* value
Male	5.9 (0.7–49)	0.040
T3-tumour	41 (2.8–606)	0.002
Tumour necrosis	6.3 (0.5–76)	0.138

OR = Odds ratio; CI = Confidence Interval

### Survival outcome

The overall 1-, 3-, and 5-year survival rates following primary nephrectomy for RCC were 94%, 86%, and 79%, respectively. Metastatic disease occurred in 31% of patients, with a mean time to detection of 25 months, and 92% diagnosed within 60 months post-surgery. Survival rates from diagnosis of metastasis for patients with secondary malignancies were 74%, 58%, and 51% at 1, 3, and 5 years, respectively (Table 4).

**Table 4:** Survival outcomes and time to secondary malignancy in renal cell carcinoma patients after nephrectomy

**Surgery for renal cell carcinoma**	n754
1-year survival	709 (94)
3-year survival	648 (86)
5-year survival	596 (79)
Time from primary surgery to secondary malignancy (months), n236 ^a^	25 (26)
**Survival outcomes following secondary malignancy after kidney cancer surgery**	236 (31)
1-year survival	174 (74)
3-year survival	137 (58)
5-year survival	120 (51)
Proportion of secondary malignancies detected within 60 months	218 (92)

The 1-, 3-, and 5-year survival rates, as well as the mean time from primary nephrectomy (radical or partial) to the development of secondary malignancies, in patients diagnosed with renal cell carcinoma (*n* = 754). The incidence of secondary malignancies is reported (*n* = 236), along with their survival outcomes and the proportion detected within 60 months of the primary surgery. ^a^ Mean value (SD)

In patients with liver or pancreatic metastases (6%), the mean time to metastasis was 25 months. Among these, patients who underwent secondary surgery (25%) showed significantly superior survival outcomes (92%, 83%, and 75% at 1, 3, and 5 years) compared to non-surgical patients (77%, 65%, and 56%), with a mean time to metastasis of 57 months versus 22 months (Table 5).

**Table 5:** Survival outcomes and time to liver or pancreas metastasis in renal cell carcinoma patients: comparison of patients without and with secondary surgery

Liver or pancreas metastasis n48	Liver or pancreas metastasis without secondary surgery n36 (75)	Liver or pancreas metastasis with secondary surgery n12 (25)
1-year survival	30 (77)	11 (92)
3-year survival	26 (65)	10 (83)
5-year survival	21 (56) ^*^	9 (75)
Time from primary surgery to liver or pancreas metastasis ^a^	22 (24)	57 (50)

This table presents the 1-, 3-, and 5-year survival rates, as well as the mean time from primary surgery to liver or pancreas metastasis, in patients with liver or pancreas metastases from renal cell carcinoma (*n* = 48). Patients are stratified by those who underwent secondary surgical intervention (*n* = 12) versus those who did not (*n* = 36). The time to metastasis is shown as a mean value with a range. ^a^ Mean value (SD). ^*^ Two patients who underwent surgery in 2020 and 2021 have been excluded from the 5-year survival analysis due to insufficient follow-up time

## Discussion

The findings of this study provide valuable insight into the predictors of liver and pancreatic metastases in patients with RCC following nephrectomy. Our results confirm the critical role of advanced tumor stage (T3-T4), in predicting the development of secondary malignancies [13, 14], specifically liver and pancreatic metastases [15]. The case group, consisting of 12 patients with liver or pancreatic metastases, exhibited significantly higher rates of local tumor invasiveness, including collecting system and perinephric fat invasion [16–18]. These findings suggest that advanced local tumor characteristics, such as local invasion, may play a pivotal role in the subsequent spread of RCC to distant organs, particularly the liver and pancreas.

Advanced tumor stage, identified as a robust predictor in our multivariate analysis, was the most consistent risk factor associated with the development of metastasis. This result aligns with prior research associating advanced tumor stage and T3-T4 disease to an increased probability for regional and distant spread [13–17, 19]. The absence of patients with T1 or T2 tumors in the metastatic group further strengthens the hypothesis that more advanced tumor stages are directly correlated with metastatic risk [19]. Previous studies have demonstrated a correlation between male gender and tumor necrosis with an increased risk of metastasis in RCC [17, 20]. In this study, these factors had an association with metastasis but did not reach statistical significance in our analysis. However, the higher frequency of tumor necrosis in the case group (33% versus 13%) may indicate a more aggressive tumor phenotype in patients who develop metastases. A key clinical implication of these findings is the strong association between advanced tumor stage and the development of metastasis, which may advise clinical decision-making and follow-up strategies. The tumors invasiveness is associated with a greater risk of secondary malignancy and supported by similar observations in RCC metastasis studies [4, 13, 14, 16, 18, 21, 22]. Adding to the growing evidence that intensive post-surgical surveillance may benefit patients with high-risk tumor features [6, 7, 23, 24]. Our study highlights the importance of vigilant surveillance in patients with T3 disease, especially in the first five years following nephrectomy.

Given that 92% of secondary malignancies were detected within 60 months post-surgery, our results support the current practice of a five-year follow-up period for these patients. However, the early onset of metastasis observed in our cohort—median time to metastasis was 25 months—suggests that more intensive and possibly earlier imaging follow-up may be beneficial, particularly within the first few years post-surgery.

Surgical resection of liver or pancreas metastases was associated with significantly improved survival outcomes [22, 25–28]. Patients who underwent surgery achieved 1-, 3-, and 5-year survival rates of 92%, 83%, and 75%, respectively, compared to 77%, 65%, and 56% for non-surgical cases. These findings brings into question whether the frequency of follow-up imaging should be adjusted, particularly for patients with advanced disease who may be at higher risk for early metastasis.

The time to the development of liver or pancreatic metastases differed substantially between patients eligible for surgical intervention (57 months) and those who were unsuitable (22 months). This finding suggests that a prolonged interval to metastasis is a favourable factor for surgical eligibility and is associated with improved overall survival. Large case series indicate that single-site metastatic disease in the pancreas is more favourable than metastasis in other sites [29]. In this study, 92% of the surgical cohort had ccRCC, a subtype known for slower progression. This may explain the extended time to metastasis observed in this group. The predominance of ccRCC could raises the question of whether patients with more aggressive RCC subtypes are less likely to undergo metastasis surgery. As a consequence, should these patients receive more extensive evaluation, perhaps using alternative diagnostic methods, to enable earlier detection and increase the likelihood of surgical intervention.

Using biomarkers or advanced imaging techniques, such as PET-CT or MRI, may help detect early signs of aggressive tumor growth and metastasis [30, 31]. These methods could therefore allow for earlier identification of tumors at higher risk of spreading, enabling prompt intervention and potentially improving outcomes. Enhanced diagnostic tools may ultimately lead to further personalized treatment strategies, improving survival for high-risk patients. Further studies are needed to assess the benefits of tailored surveillance for high-risk RCC subtypes to improve surgical eligibility for liver and pancreatic metastasis. We identified limitations in this study. As a retrospective study, there is a risk of confounding including potential selection biases in decisions regarding secondary surgery. The 5-year survival rate could not be calculated for two patients in the group with liver or pancreatic metastases who did not undergo secondary surgery and had their primary surgery in 2020 and 2021, as their follow-up periods remain incomplete. Although these patients were included in the overall cohort, their exclusion from the survival analysis may lead to an underestimation of the 5-year survival rate. Furthermore, the small sample size of the metastatic group limits both the statistical power and the generalizability of our findings, and results should be interpreted with caution. Due to the small sample size and the risk of further reducing statistical power, no matching between cases and controls was performed. Instead, multivariable logistic regression and bootstrap analyses were used to adjust for potential confounding. The strengths of this study included a well-defined cohort, and a comprehensive analysis of clinical, radiological, and pathological factors associated with liver and pancreatic metastasis in RCC. The use of multivariable logistic regression combined with bootstrap analysis tailored to the small sample size enhanced the robustness of our findings, particularly the observed association between advanced tumor stage and metastasis. Larger studies are needed to validate our findings.

## Conclusions

In this study, advanced tumor stage and local invasiveness were key predictors of metastasis. The prognostic significance of prolonged time to liver or pancreas metastasis was emphasized, with longer interval favouring surgical eligibility and improved survival in RCC patients post nephrectomy. Surgical resection of isolated metastases has been shown to significantly improve survival in selected patients. Notably, the majority of patients eligibility for metastasis surgery had ccRCC, a subtype known for its slower progression. This raises the question of whether patients with more aggressive forms of RCC are less likely to be considered for surgery. These findings highlight the importance of further studies to investigate whether early, individualized follow-up and intervention strategies, particularly for high-risk RCC subtypes, could improve surgical eligibility for liver and pancreatic metastasis.

## Electronic supplementary material

Below is the link to the electronic supplementary material.

Supplementary Material 1

## Data Availability

Data is provided within the manuscript or supplementary information files.
