# Supplementary material for: Survival outcomes and risk factors for liver and pancreatic metastases in renal cell carcinoma after curative nephrectomy
Source: BMC Urol. 2025 May 15;25:123. doi: 10.1186/s12894-025-01802-x (PMC12079807; doi:10.1186/s12894-025-01802-x)
Supplement: Supplementary file 1 — Supplementary Material 1 [file 12894_2025_1802_MOESM1_ESM.docx]

**Supplementary file**

**Supplementary Table 1.**

| **Bootstrap for Variables in the Equation** | | | | | | | |
| --- | --- | --- | --- | --- | --- | --- | --- |
|  | | B | Bootstrap^a^ | | | | |
|  |  |  | Bias | Std. Error | Sig. (2-tailed) | 95% Confidence Interval | |
|  |  |  |  |  |  | Lower | Upper |
|  | Stage T3 | 3,716 | 13,704^b^ | 14,340^b^ | ,002^b^ | 1,942^b^ | 55,426^b^ |
|  | Gender | 1,781 | 4,209^b^ | 8,340^b^ | ,040^b^ | -,178^b^ | 21,963^b^ |
|  | Tumour necrosis | 1,837 | 7,486^b^ | 13,057^b^ | ,138^b^ | -1,739^b^ | 39,637^b^ |
|  | Constant | -4,971 | -17,479^b^ | 18,100^b^ | ,001^b^ | -73,621^b^ | -2,842^b^ |
| a. Bootstrap results are based on 5000 bootstrap samples | | | | | | | |
| b. Based on 4991 samples | | | | | | | |
